# Update on the Pathogenesis of Enteropathy-Associated T-Cell Lymphoma

**DOI:** 10.3390/diagnostics13162629

**Published:** 2023-08-09

**Authors:** Shahed Azzam Ahmed Abdullah, Patricia Goa, Elisabeth Vandenberghe, Richard Flavin

**Affiliations:** 1 Department of Histopathology, Trinity College Dublin, D02 PN40 Dublin, Ireland; 2 Department of Histopathology, St. James’s Hospital, D08 NHY1 Dublin, Ireland; pgoa@stjames.ie; 3 Department of Haematology, St. James’s Hospital, D08 NHY1 Dublin, Ireland; evandenberghe@stjames.ie; 4 Department of Haematology, Trinity College Dublin, D02 PN40 Dublin, Ireland

**Keywords:** EATL, T-cell non-Hodgkin lymphoma, intraepithelial T-lymphocytes, coeliac disease, genetics, refractory coeliac disease, small intestine

## Abstract

EATL is an aggressive T-cell non-Hodgkin lymphoma with poor prognosis and is largely localized to the small intestine. EATL is closely associated with coeliac disease (CD) and is seen mostly in patients originating from Northern Europe. Various factors are associated with an increased risk of developing EATL, such as viral infection, advanced age, being male, and the presence of the HLA-DQ2 haplotype. Clonal rearrangements in the TCR-β and γ genes have been reported in all EATL morphological variants with distinctive immunophenotypic characteristics. Although EATL can occur de novo, individuals with RCDII are at a higher risk of developing EATL. The cells of origin of EATL has been postulated to be normal small intestinal intraepithelial T-lymphocytes (IELs), and more recent evidence suggests a link between innate precursor IELs and EATL derived from refractory coeliac disease type II (RCDII). The immune microenvironment of mucosal cells within the small intestine enhances the process of neoplastic transformation of IELs into EATL. Cytokines such as IL-15 can activate and crucially deregulate the JAK-STAT signaling pathway by binding to receptors on the surface of IELs. Furthermore, mutations in the JAK/STAT pathway have been associated with RCDII-derived EATL.

## 1. Introduction

**Figure 1:**
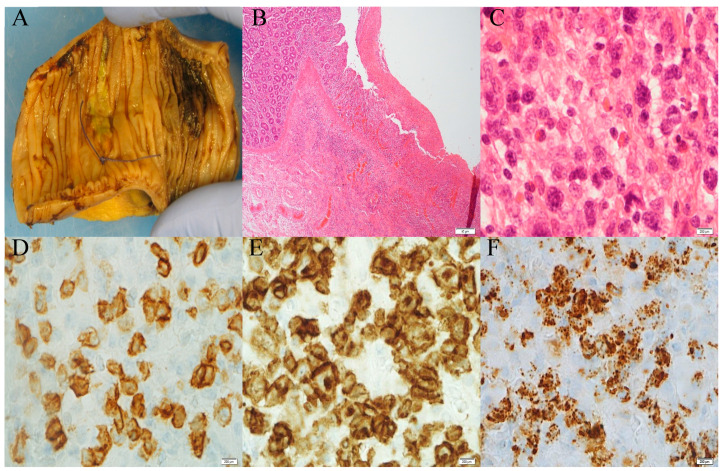
(**A**) Gross photograph of the small bowel with ulceration and haemorrhage from a patient with known coeliac disease. (**B**) Microscopy of ulceration and adjacent total villous atrophy increase intraepithelial lymphocytes and mural infiltration by atypical lymphoid cells (magnification: 4×). (**C**) Atypical lymphoid cells composed of medium to large cells with angulated nuclei, prominent nucleoli, and clear to eosinophilic cytoplasm (magnification: 100×). The cells are positive for CD3 (**D**), CD7 (**E**), and cytotoxic protein TIA1 (**F**).

Enteropathy associated T-cell lymphoma (EATL) is an aggressive T-cell non-Hodgkin lymphoma derived from the malignant transformation of intestinal intraepithelial lymphocytes (IELs), and is considered the most common neoplastic complication of coeliac disease (CD) [1]. EATL was reclassified in 2016 and separated from a similar intestinal lymphoma (Figure 1), monomorphic epitheliotropic intestinal T-cell lymphoma (MEITL). Type I (now classified as EATL) is frequently associated with CD and observed in Northern Europe, and Type II, now classified as MEITL, occurs de novo and is predominant in Asia [2,3]. Due to the rarity of EATL, information is scarce about the precise mechanisms underlying its pathogenesis. However, whilst genetic and environmental risk factors play an important role, an inflammatory cytokine-rich microenvironment and genotoxic stresses, including the JAK-STAT pathway, have a profound impact on lymphomagenesis.

Although EATL may emerge ‘de novo’, refractory coeliac disease Type II is strongly associated with the development of EATL as an intermediate stage in neoplastic progression (in contrast to Type I) [2,4]. RCDII can be defined as unresponsiveness to a strict gluten-free diet (GFD) for at least a period of 6–12 months and is associated with an aberrant IEL phenotype [2,3]. EATL pathogenesis is complex and multifactorial with limited research currently regarding the precise mechanisms of malignant transformation. There is a great need to further classify the cell of origin and identify the various mutations facilitating neoplastic transformation to EATL. In this review, we aim to provide an update regarding the genetic and environmental risk factors as well as the molecular pathogenesis of EATL.

## 2. Establishment of Clinical Diagnosis

As there is a link between EATL and coeliac disease (CD), the pathogenesis of CD will first be briefly outlined. CD is an autoimmune disease of the small intestine caused by exposure to gluten in a genetically susceptible individual [5]. Gluten, which is the protein component of grains such as wheat, rye, and barley, is the trigger of CD [5]. CD presents with signs and symptoms of malabsorption, such as chronic recurrent diarrhea, steatorrhea, abdominal pain, abdominal distention, and unexplained weight loss [5]. These are classical symptoms, but studies have shown that many patients present atypically with symptoms of anemia and/or complications such as small intestinal ulceration/haemorrhage (Figure 1A) [5]. The genetically predisposing factors most extensively studied in CD patients are HLA-DQ2 and/or HLA-DQ8, which are identified in almost 90–95% of patients. Ninety percent express HLA-DQ2, and the remaining 10% express HLA-DQ8 [5]. Furthermore, poor adherence to a GFD, HLA-DQ2 homozygosity, and late diagnosis of CD are recognized as risk factors for the malignant evolution of CD [5].

Serologically, there are various markers of significance for CD. The first set of markers is auto-antibodies against autoantigens, such as IgA anti-tissue transglutaminase (anti-tTG-ab), and anti-endomysial antibodies (EMA) [5]. The second set of markers are antibodies against gliadin, antigliadin IgA (AGA), and anti-deamidated forms of gliadin peptide antibodies (DGP) [5]. The sensitivity and specificity of these markers are very high—except AGA, as there is increased prevalence of IgA deficiency in CD patients [5]. Even though these markers are very specific and sensitive, upper endoscopy with a small bowel biopsy plays a critical role by showing evidence of villous atrophy and is an essential prerequisite for the diagnosis of CD [5]. Biopsies from the duodenum might show a range of features, such as a mild increase in intraepithelial lymphocytes (Marsh 1), increased intraepithelial lymphocytes with crypt hyperplasia (Marsh 2), or either of the prior features with varying degrees of villous atrophy (Marsh 3) [6]. To make a diagnosis of coeliac disease, there needs to be a Marsh score of ≥2, with serological markers and a response to a gluten-free diet [6].

As a known complication of CD, EATL is derived from the neoplastic transformation of aberrant IELs emerging in patients with CD that are not responding to GFD [7]. An extensive clinical diagnostic workup involves magnetic resonance enteroclysis, a positron emission tomography scan, and a histologic identification of lesions present [7]. RCDI is characterized by a persistence of villous atrophy, despite a strict GFD and increased but phenotypically normal IELs [7]. In contrast, in RCDII cases, there is a clonal expansion of abnormal IELs lacking surface markers such as CD3, CD8, and T-cell receptor (TCR) but expressing intracellular CD3 [7]. 

A histologic examination of small intestinal biopsies remains crucial in the diagnostic workup of EATL [7]. Morphologically, in EATL cases, there is frequently medium- to large-cell or pleomorphic cytology consisting of angulated vesicular nuclei, prominent nucleoli, and a pale-staining cytoplasm, as well as an increased mitotic index, often associated with a moderate to abundant reactive milieu of eosinophils, histiocytes, and small lymphocytes (Figure 1C). Immunohistochemically neoplastic cells are typically CD3^+^ (Figure 1D), CD7^+^ (Figure 1E), CD103^+^, TCRβ^+/−^ cells, CD4^−^, CD8^−^, and CD5^−^, mostly expressing CD30 with CD56 negativity [8], and demonstrate an activated cytotoxic phenotype (perforin^+^, granzyme B^+^, and TIA-1^+^) (Figure 1F), reflecting the origin of the tumor from cytotoxic IELs [8]. Adjacent mucosa can show the histological features of active CD, including increased IEL infiltration, crypt hyperplasia, and villous atrophy (Figure 1B) [8]. 

Conversely, MEITL is less frequently associated with coeliac sprue, as is the HLA-DQ2/HLA-Dq8 haplotype [7], and is characterized by a monomorphic infiltrate of small- to medium-sized lymphoid cells expressing CD3^+^, CD4^−^, CD8^+^, CD56^+^, and TCRβ^+^ with CD30 negativity associated with an absence of villous atrophy [8]. CD56 positivity likely suggests a different underlying mechanism of the lymphomagenesis process in MEITL [8]. The morphology and immunophenotype of MEITL are distinct from those of EATL, which shows striking infiltration of the intestinal epithelium and lacks the inflammation and necrosis that are characteristic of EATL [9]. 

It is proposed that PCR analysis has a high predictive value and aids in identifying RCD2, via the detection of monoclonal rearrangements of the TCR-γ chain in intestinal tissue sections, supplemented by adjunct immunohistochemistry or flow cytometry, which further helps in the characterization of the aberrant T-cell IEL population [8]. Duodenal biopsies of patients with pre-malignant RCDII showing TCR-γ clonal amplification had similar clonal profiles detected in subsequent EATL tumor specimens [8]. 

## 3. Genetics and Environmental Factors

Risk factors for EATL include being male, advanced age, and both genetic and environmental factors. EATL shows a slight predominance in men (54%), most of whom are older than 50 years of age [7]. One previously hypothesized environmental trigger was the theory of early weaning, with an early introduction of gluten into the infant’s diet being associated with the high incidence of CD in pre-disposed infants [10]. Recent studies, however, show no evidence that the delayed weaning or early introduction of gluten itself will impact the likelihood of developing CD in the future, even among infants with genetic susceptibility. Hence, holding off gluten introduction does not have any advantage in predisposed populations [10]. Although the duration of gluten diet exposure plays an important role in EATL pathogenesis via the enhancement of inflammatory signaling pathways mediated by CD4^+^ T-cells, many cases of EATL arise despite a strict GFD [11,12].

Genetic susceptibility exists in the form of HLA-DQ2/HLA-DQ8 haplotypes [13]. Furthermore, there is a strong correlation between HLA-DQ2.5 homozygosity and the development of EATL [14]. In one systematic review, HLA-DQ2.5 homozygosity was found in 53.3% of patients with EATL and 44.1% of patients with RCDII [14]. A genome-wide association study (GWAS) reported a strong association between SNP Chr7p14.3 and progression to RCDII as a result of alteration in Paneth cell genes [15]. Innate immune Paneth cell dysregulation and intestinal microbiota play a role, as reported by Di Sabatino et al. [16]. In addition, the presence of SNP rs7259292 in the MYO9B gene on chr9 is another genetic risk factor for the development of RCDII and its progression to EATL [16,17]. EATL is characterized genetically by chromosome 9q31.3 gain or 16q12.1 deletion, whilst MEITL is characterized by chromosome 8q24 gain and, less commonly, by 1q and 5q gains [7].

The role of environmental risk factors is poorly understood, but recent studies have reported an associated between EATL and infection with EBV, which is thought to drive chronic inflammation and mediate T-cell cytotoxicity [18]. Specifically, EBV could contribute to viral-induced chronic inflammation by mediating and enhancing T-cell cytotoxicity from circulating EBNA-1-reactive CD8^+^ T-cells, thereby augmenting EATL development [18]. EBV positivity within inflammatory cells and enterocytes was detected in 70.5% of patients with RCDII [18]. Further studies are still needed to determine the strength of the association between EBV and EATL pathogenesis [18].

## 4. Molecular Pathogenesis

### 4.1. Cell of Origin

Anatomically, EATLs most commonly arise in the jejunum or ileum (90%) as well as the large intestine and stomach, subsequently infiltrating the mesenteric lymph nodes [7]. However, they may also be located extra-intestinally in the lung (5%), skin (5%), and bone marrow (3%) [7]. When located in the small intestine, EATLs present most often as single or multiple mucosal lesions or tumor masses and may present as ulcerative jejunitis [19]. The expression of the enterocyte marker NKp46 on aberrant IELs in the presence of IL-15 confers the tumor with the ability to carry out enterocyte killing, resulting in severe ulceration [20]. Additionally, the angiocentricity and angioinvasion displayed by these cells may lead to extensive necrosis, often resulting in obstruction, intestinal perforation, and peritonitis [19]. 

Intestinal IELs are a set of heterogeneous T-lymphocytes, natural killer (NK) precursors, and innate or immature T-cells [21]. Aberrant IELs originate from deranged immature T-lymphocytes and display clear differentiation to a cytotoxic phenotype [21]. Aberrant IELs displayed different stages of maturity in individuals with RCDII, and only those harboring the most mature aberrant IELs clonal population develop EATL [21]. 

EATL arises from malignant transformation either de novo as a complication of CD or secondary to RCDII, and is characterized by the clonal population expansion of aberrant IELs [22]. These aberrant IELs arise from innate CD34^−^, TCR^−^, CD7^+^, and sCD3^−^IELs within the small intestine normal mucosa [20]. Innate iCD3^+^ IELs phenotypically show the same features as the clonal expansion of aberrant IELs seen in cases of RCDII and EATL, which indicates that they are the cells of origin [20]. 

In response to NOTCH signals, iCD3^+^ innate IELs arising from lymphoid precursors within the gut epithelium undergo T-cell differentiation and reprogram into innate-like cells or NK-like cells in the presence of IL-15 [20]. Subsequently, IL-15 reprograms these lymphoid precursors by inducing the granzyme B (GrB)-dependent degradation of NOTCH1 [6]. Ultimately, the production of IFN-γ is induced following the expression of CD122, T-BET, and GrB on RCDII IELs via an NK receptor (notably NKp46) [20,21,22]. These features allow the aberrant IELs to carry out NK-like cytotoxicity against epithelial cells [20]. 

In RCDII, the majority of T-cell receptor gene rearrangements occur as out-of-frame *TCRG*, *TCRD*, and *TCRB* rearrangements, which further highlights that the aberrant IELs begin to undergo T-cell differentiation but do not complete it [20]. The accumulation of mutations as a result of rearrangements of clonal TCR genes and the expression of intracellular CD3ε and CD3γ within the cytokine-rich environment characterize the T-cell features of aberrant IELs leading to malignant transformation [20]. 

The further accumulation of mutations in a cytokine-rich environment results in the malignant transformation of iCD3-innate IELs, which display all of the phenotypic features of aberrant clonal IELs in cases of RCDII and EATL. This certifies that these iCD3^+^ IELs are the cells of origin of RCDII and EATL [20].

### 4.2. Cytokine Signaling

Coeliac disease is an autoimmune disease of the small intestine caused by exposure to gluten in genetically susceptible individuals [23]. Gluten is composed of gliadin and is the major trigger of coeliac disease [23]. The IgA-coated gliadin interacts with HLA-DQ2 or DQ8 CD4^+^ T-cells within the mucosa, resulting in the activation of CD4^+^ T-cells, and this produces pro-inflammatory cytokines such as Th-1- and Th-2-derived cytokines [23]. The Th-1 response produces IFN-γ, while the Th-2 response results in the clonal expansion of B-cells, the formation of plasma cells, and the release of anti-tissue transglutaminase (Anti-tTG) and anti-gliadin antibodies [20,21,22,23]. The anti-tTG enzyme plays a role in the process of the deamidation of glutamine in gluten to glutamic acid, resulting in negatively charged gluten [23]. The activation of T-cells has been associated with increased binding of negatively charged gluten to HLA-DQ2 and DQ8, which further increases their ability to activate more T-cells [20,21,22,23]. 

RCDII is characterized by unresponsiveness to a GFD, providing a chronic inflammatory environment, which promotes genotoxic stress, ultimately leading to malignant transformation [17,20]. Interleukin-15 (IL-15) stimulates the expression of various cytotoxic proteins and impairs the negative regulation of RCDII IELs via T-regulatory cells and TFG-β [24]. Furthermore, IFN-γ production and their NKG2D-dependent cytotoxic activity against enterocytes are stimulated via IL-15 [24,25]. The mechanism of this uncontrolled chronic antigenic stimulation is facilitated by the IL-15Rα, which can bind IL-15 and form enduring complexes at the cell membrane, subsequently activating RCDII IELs [24,25]. This cascade mechanism potentiates an inflammatory response and promotes the genomic instability of the aberrant cell population [26]. 

Epithelial-derived IL-15 plays an important role in the expansion of aberrant cell population and its subsequent conversion to EATL. Chronic inflammation-related genotoxic stress in RCDII cases promotes an environment in which aberrant IELs may gain and gradually accrue genetic aberrations and mutations [24,27,28]. The acquisition of these mutations by IELs allows them to progressively expand at the expense of other IELs, as JAK1 or STAT3 gain-of-function mutations garner the cells with hyper-responsiveness to IL-15 [20]. The ability of aberrant IELs to outcompete normal polyclonal IELs is coupled with the anti-apoptotic function of IL-15. This facilitates the emergence of a clonal proliferation of aberrant IELs, which ultimately acquire additional mutations during their expansion, hence promoting the transformation into EATL. Furthermore, the uncontrolled overexpression of IL-15 in RCDII cases facilitates lymphomagenesis [20].

Interestingly, only 40% of patients with active CD display an upregulation of IL-15, with a substantial proportion of aberrant IELs expressing low levels of CD122 [29]. This finding highlights the importance of the role of cytokines as part of the adaptive immune response in driving aberrant IEL proliferation. The cytokines IL-2, IL-21, and TNF produced by gluten-specific CD4^+^ T-cells act synergistically to influence the proliferation of aberrant IELs [11]. These cytokines increase the phosphorylation of STAT5 and Akt and the transcription of Bcl-xL, thereby confirming the association of HLA-DQ2 with RCDII [11]. IELs lacking TCR/CD3 surface expression characterize RCDII [29]. Although RCDII IELs exhibit several chromosomal abnormalities, they fail to proliferate at a high rate [11]. This suggests that their progressive accumulation is due to failed apoptotic mechanisms facilitated by the overexpression of IL-15 [11]. 

**Figure 2:**
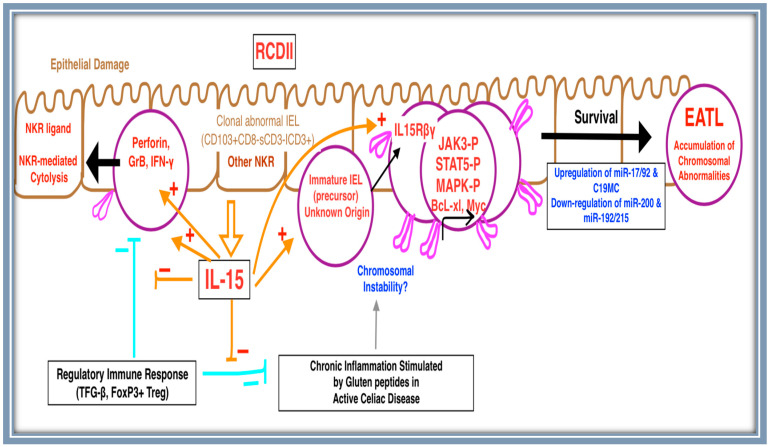
Schematic diagram illustrating the proposed EATL pathogenesis: RCDII is characterized by the progressive expansion of IELs inheriting an aberrant T/NK phenotype associated with chromosomal abnormalities. The clonal expansion of abnormal IELs occurs within the *lamina propria* and transforms into EATL through a variety of mechanisms. IL-15, produced by enterocytes, plays a central role in RCDII transformation into EATL. IL-15 is proposed to be the initiating trigger acting on abnormal immature lymphoid precursors. One of the mechanisms proposed is the IL-15 activation of NK-like RCDII IEL cytotoxicity against epithelial cells through NK receptors expressed on RCDII IELs (pink color) and their corresponding ligands expressed on epithelial cells. This results in damage to the epithelial cells. The apoptosis of RCDII IELs is prevented via a plethora of IL-15-mediated pathways involving JAK3, STAT5, MAPK, c-Myc, and the anti-apoptotic factor BCL-xL. In addition, IL-15 plays a role in the blockage of regulatory pathways resulting in prolonged chronic inflammation, which further promotes chromosomal instability. Ultimately, the survival of RCDII IELs is facilitated by the upregulation of miR-17/92 and C19MC and the downregulation of miR-200 and miR-192/215 families via c-Myc overexpression, potentiating EATL development.

Therefore, IL-15 mediates its anti-apoptotic cascade via JAK3 and STAT5 phosphorylation and Bcl-xL expression in IELs of RCDII [28]. Ultimately, this results in the inhibition of elimination and enables the acquisition of mutations and malignant transformation into EATL [28]. Hence, the overexpression of uncontrolled IL-15 plays an important role in RCDII pathogenesis and its progression into EATL (Figure 2) [28]. Interestingly, Schmitz et al. report that contact with dendritic cells (DCs) in the epithelium contributes to the uncontrolled expansion of aberrant IELs of RCDII, independently of IL-15 and the HLA genotype [30]. 

### 4.3. Mutational Landscape

The JAK/STAT pathway is considered to be among the most commonly mutated signaling pathways in EATL pathogenesis [31,32]. Recent findings show evidence linking recurrent activating mutations in members of the JAK/STAT pathways to the development of EATL and RCDII [27,31]. This evidence suggests cytokine signaling deregulation to be one of the earliest events in the process of lymphomagenesis [27]. The majority of JAK/STAT mutations are gain-of-function mutations [33]. In contrast, mutations in the negative regulator of JAK/STAT, SOCS1, occurring when JAK1 or STAT3 mutations are absent, have been reported as deletions [32]. 

In addition, EATL is characterized by the overexpression of the IFN-γ signaling pathway genes [32]. Mutations in RCDII cell lines have also been detected in association with negative regulators of NF-kB, such as TNIP3 and TNFAIP3, reported as copy number variations [32]. While these mutations do not occupy a primary role in lymphomagenesis, mutations activating JAK1-STAT3 may synergize with mutations activating NF-kB (Table 1) [32,33].

Interestingly, the most frequently mutated gene in MEITL is the *SETD2* tumor suppressor gene (TSG) [31], found in 93% of cases, serving as an important diagnostic marker (Table 1) [34]. The majority of *SETD2* mutations are a loss of function, exhibiting frameshift or nonsense mutations [28]. According to Roberti et al., *SETD2* was found to be the most significantly recurrent mutating gene exhibiting a loss of function or a loss of a corresponding locus on chromosome 3p21.31 in greater than 90% of the cases examined by whole-exome sequencing (WES) [34]. 

Other less commonly involved mutations include SOCS1 [31]. MAPK pathway mutations include KRAS, BRAF, NRAS, and DNA repair TSGs such as TP53 [31]. Soderquist et al. recently showed mutations involving epigenetic regulators such as TET2, KMT2D, the NF-κB gene TNFAIP3, the DNA damage repair gene POT1, and immune evasion pathway genes involving CD58 [22]. The JAK1-STAT3 pathway is a potential therapeutic target in RCDII cases.

Manso et al. analyzed a series of EATL and MEITL cases in order to identify specific therapeutic targets utilizing gene expression and mutational studies in formalin-fixed paraffin-embedded tissues. Most EATL patients showed mutations in DNA repair genes (TP53), followed by NOTCH, VEGF, or PI3K/AKT signaling pathways. Conversely, mutations in the *SETD2* gene, the RAS gene, or the JAK/STAT pathway were found in MEITL cases [35]. Additionally, RCDII lines were used in preclinical models in vitro to assess for the therapeutic efficacy of drugs targeting JAK2-STAT3 gain-of-function mutations using ruxolitinib, which inhibits JAK1 and JAK2 along with abrocitinib, which specifically inhibits JAK1 [36]. The effect of these two drugs was compared with the corticosteroid budesonide and the proteasome inhibitor bortezomib, which can interfere with the STAT3 signaling pathway [37]. Both ruxolitinib and abrocitinib induced apoptosis, reduced proliferation, and, simultaneously, inhibited STAT3 phosphorylation in all four RCDII-cell tested lines [36]. Hence, in RCDII cases, the JAK1-STAT pathway is a potential therapeutic target that may prevent the neoplastic progression of RCDII to EATL and improve the prognosis of the disease. 

### 4.4. MicroRNA

miRNAs are a class of noncoding RNAs involved in differentiation, programmed cell death, cellular growth, and tumorigenesis. The potential role of miRNAs as TSGs involved in signaling pathways such as JAK/STAT is a major cornerstone in the process of the lymphomagenesis of aberrant IELs [38]. The association between 13 downregulated miRNAs in EATL samples and the JAK/STAT signaling pathway have been reported in a pilot study conducted by Clarke et al. [39]. These miRNAs act as potential tumor suppressor genes [39]. Notably, the 13 identified miRNAs specifically targeted the JAK/STAT pathway at a high rate [39]. In cases of EATL, many of the miRNAs are located within regions involved in chromosomal aberrations [40]. 

miRNAome analysis has shown that miR-200 and miR-192/215 families are progressively lost in both RCDII and EATL. In contrast, the oncomiRNA families miR17/92 and C19MC are upregulated [40]. The upregulation of miR-17/92 and C19MC occurs via increased nuclear SMAD3, MDM2, and activated STAT3 [22]. Additionally, the downregulation of the miR-200 and miR-192/215 families is observed via c-MYC overexpression [40]. While not every case of EATL exhibits an upregulation of the miR17/92 cluster, those exhibiting the oncogenic feedback loop associated with c-Myc overexpression have a worse prognosis, enabling the lymphomagenesis sustainability of aberrant IELs [40]. 

The role of miRNA deregulation is a crucial factor in the process of the neoplastic transformation of aberrant IELs observed in cases of RCDII into EATL [34]. This is evident by the c-Myc overexpression observed in EATL cases and the activation of the STAT3 signaling pathway upstream of c-Myc in RCDII cases [40]. miRNAs may also provide clinicians with a novel method of classifying these disease entities. While RCDII retains many similar characteristics to EATL, it must be recognized as a distinct disease. Accordingly, RCDII was characterized by a specific miRNA profile, that is more similar to CD and RCDI than to EATL, thereby highlighting the specific characteristics of EATL [40].

## 5. Conclusions

Mature T- and NK-cell neoplasms collectively represent less than 15% of all NHL cases. This paper mainly focused on the pathogenesis of EATL as a rare and rapidly fatal intestinal T-cell NHL [41]. EATL has poor prognosis akin to other subtypes of T-NHLs such as T-cell lymphoblastic lymphoma, anaplastic large cell lymphoma, adult T-cell lymphoma/leukemia, angioimmunoblastic T-cell lymphoma, extranodal NK/T-cell lymphoma, and peripheral T-cell lymphoma (not otherwise specified) [41]. The pathogenesis of EATL is not fully understood, but recently many advances have contributed to the understanding of EATL lymphomagenesis. Genetic susceptibility exists in the form of HLA-DQ2/HLA-DQ8 haplotype expression. 

Intraepithelial T-cells are presumed to be the cells of origin of RCDII and EATL. The characterization of RCDII as an intermediate stage in the stepwise pathogenesis of EATL has provided scope for the examination of the mechanisms by which cytokine signaling and chronic antigenic stimulation contribute to the emergence of a malignant proliferation. This is underpinned by an understanding of the mutational landscape of EATL and the central role of the JAK/STAT signaling pathway in favoring the emergence of malignancy. 

EATL displays several chromosomal alterations, which accumulate over the course of RCDII due to its genotoxic environment and subsequently facilitate transformation to EATL. Furthermore, inflammatory signaling acts synergistically with the aberrant chromosomal and mutational landscape of EATL to promote neoplastic proliferation. Elevated levels of IL-15 in the small intestine of CD and RCD patients likely contribute to the expansion of aberrant IELs observed in RCDII cases and the progression to EATL. 

Recent GWAS studies, despite their limitations, may provide an encouraging avenue into elucidating the pathogenesis of EATL. The evaluation of the miRNAome has proved promising. Overexpression of the c-MYC-regulated miR-17/92 cluster distinguishes MEITL from EATL and further prognosticates EATL outcomes [34,42]. Clinicians may utilize miRNAs as a novel method of disease classification and treatment optimization. 

Ultimately, there is a critical need to advance our understanding of EATL for the further optimization of diagnostic and therapeutic options, as illustrated in Table 1. This is particularly critical in populations with a high incidence of CD and the associated high mortality rate as a result of malignant transformation to EATL. Additionally, there is a need to further evaluate the strength of the protective effects of gluten-free diets on EATL via prospective cohorts and experimental studies [43].

## Figures and Tables

**Table 1:** Frequency of various mutational profiles of RCDII-EATL and de novo EATL.

Pathway	Somatic Mutations	Primary Mechanisms of Mutation	RCDII-EATL Mutation Frequency	De Novo EATL Mutation Frequency	Clinical Significance
JAK/STAT	JAK1 [32] STAT3 [32] SOCS1 [32] SOCS3 [32]	Gain-of-function mutations	48% 38% 7% 8%	32% (double mutation with STAT3) 32% (double mutation with JAK1)	Therapeutic and potential diagnostic value [32]
NF-κB	TNFAIP3 [32] TNIP3 [32]	Nonsense or frameshift mutations	13% 9%	28%	Potential prognostic value [32]
Gene Regulation	KMT2D [32] TET2 [32] POT1 [32]	Loss of function (frameshift, nonsense, or missense mutations)	22% 30%	37% 32% 26%	Potential prognostic value [32]
	SETD2 [31]	Frameshift or nonsense		10–20%	Diagnostic Value [31]
Gene Expression	DDX3X [32]	Missense mutation	20%	32%	Potential prognostic value [32]

## Data Availability

Not applicable.

## References

[1] Ferreri A.J., Zinzani P.L., Govi S., Pileri S.A. (2011). Enteropathy-associated T-cell lymphoma. Crit. Rev. Oncol..

[2] Tomita S., Kikuti Y.Y., Carreras J., Kojima M., Ando K., Takasaki H., Sakai R., Takata K., Yoshino T., Bea S. (2015). Genomic and immunohistochemical profiles of enteropathy-associated T-cell lymphoma in Japan. Mod. Pathol..

[3] Fei F., Reddy V., Patel C.R., Dhall D., Lee G., Meng-Jun X., Al Diffalha S. (2020). Monomorphic Epitheliotropic Intestinal T-cell Lymphoma: A Study of Four Cases and Review of Literature. Ann. Clin. Lab. Sci..

[4] Ritter J., Zimmermann K., Jöhrens K., Mende S., Seegebarth A., Siegmund B., Hennig S., Todorova K., Rosenwald A., Daum S. (2018). T-cell repertoires in refractory coeliac disease. Gut.

[5] Mir B.A., Majeed T., Singh A., Rajput M.S., Kumar A., Chauhan A. (2022). Emerging Biomarkers for Screening and Management of Celiac Disease. BioMed Res. Int..

[6] Brown J.R.G., Singh P. (2018). Coeliac disease. Paediatr. Int. Child Health.

[7] Delabie J., Holte H., Vose J.M., Ullrich F., Jaffe E.S., Savage K.J., Connors J.M., Rimsza L., Harris N.L., Müller-Hermelink K. (2011). Enteropathy-associated T-cell lymphoma: Clinical and histological findings from the International Peripheral T-Cell Lymphoma Project. Blood.

[8] Di Sabatino A., Biagi F., Gobbi P.G., Corazza G.R. (2012). How I treat enteropathy-associated T-cell lymphoma. Blood.

[9] de Leval L., Feldman A.L., Pileri S., Nakamura S., Gaulard P. (2023). Extranodal T- and NK-cell lymphomas. Virchows Arch..

[10] Silano M., Agostoni C., Sanz Y., Guandalini S. (2016). Infant feeding and risk of developing celiac disease: A systematic review. BMJ Open.

[11] Kooy-Winkelaar Y.M.C., Bouwer D., Janssen G.M.C., Thompson A., Brugman M.H., Schmitz F., de Ru A.H., van Gils T., Bouma G., van Rood J.J. (2017). CD4 T-cell cytokines synergize to induce proliferation of malignant and nonmalignant innate intraepithelial lymphocytes. Proc. Natl. Acad. Sci. USA.

[12] Askling J., Linet M., Gridley G., Halstensen T.S., Ekström K., Ekbom A. (2002). Cancer incidence in a population-based cohort of individuals hospitalized with celiac disease or dermatitis herpetiformis. Gastroenterology.

[13] Al Somali Z., Hamadani M., Kharfan-Dabaja M., Sureda A., El Fakih R., Aljurf M. (2021). Enteropathy-Associated T cell Lymphoma. Curr. Hematol. Malign-Rep..

[14] Al–Toma A., Goerres M.S., Meijer J.W., Peña A.S., Crusius J.B.A., Mulder C.J. (2006). Human Leukocyte Antigen–DQ2 Homozygosity and the Development of Refractory Celiac Disease and Enteropathy-Associated T-Cell Lymphoma. Clin. Gastroenterol. Hepatol..

[15] Hrdlickova B., Mulder C.J., Malamut G., Meresse B., Platteel M., Kamatani Y., Ricaño-Ponce I., van Wanrooij R.L., Zorro M.M., Bonder M.J. (2018). A locus at 7p14.3 predisposes to refractory celiac disease progression from celiac disease. Eur. J. Gastroenterol. Hepatol..

[16] Di Sabatino A., Miceli E., Dhaliwal W., Biancheri P., Salerno R., Cantoro L., Vanoli A., De Vincenzi M., Blanco C.D.V., MacDonald T.T. (2008). Distribution, Proliferation, and Function of Paneth Cells in Uncomplicated and Complicated Adult Celiac Disease. Am. J. Clin. Pathol..

[17] Wolters V.M., Verbeek W.H., Zhernakova A., Onland–Moret C., Schreurs M.W., Monsuur A.J., Verduijn W., Wijmenga C., Mulder C.J. (2007). The MYO9B Gene Is a Strong Risk Factor for Developing Refractory Celiac Disease. Clin. Gastroenterol. Hepatol..

[18] Perfetti V., Baldanti F., Lenti M.V., Vanoli A., Biagi F., Gatti M., Riboni R., Dallera E., Paulli M., Pedrazzoli P. (2016). Detection of Active Epstein–Barr Virus Infection in Duodenal Mucosa of Patients With Refractory Celiac Disease. Clin. Gastroenterol. Hepatol..

[19] Foukas P.G., Bisig B., de Leval L. (2021). Recent advances in upper gastrointestinal lympho-mas: Molecular updates and diagnostic implications. Histopathology.

[20] Ettersperger J., Montcuquet N., Malamut G., Guegan N., Lastra S.L., Gayraud S., Reimann C., Vidal E., Cagnard N., Villarese P. (2016). Interleukin-15-Dependent T-Cell-like Innate Intraepithelial Lymphocytes Develop in the Intestine and Transform into Lymphomas in Celiac Disease. Immunity.

[21] Tack G.J., van Wanrooij R.L., Langerak A.W., Tjon J.M., von Blomberg B.M.E., Heideman D.A., van Bergen J., Koning F., Bouma G., Mulder C.J. (2012). Origin and immunophenotype of aberrant IEL in RCDII patients. Mol. Immunol..

[22] Soderquist C.R., Lewis S.K., Gru A.A., Vlad G., Williams E.S., Hsiao S., Mansukhani M.M., Park D.C., Bacchi C.E., Alobeid B. (2021). Immunophenotypic Spectrum and Genomic Landscape of Refractory Celiac Disease Type II. Am. J. Surg. Pathol..

[23] Björck S., Lindehammer S.R., Fex M., Agardh D. (2015). Serum cytokine pattern in young children with screening detected coeliac disease. Clin. Exp. Immunol..

[24] Malamut G., Cording S., Cerf-Bensussan N. (2019). Recent advances in celiac disease and refractory celiac disease. F1000Research.

[25] Malamut G., Meresse B., Cellier C., Cerf-Bensussan N. (2012). Refractory celiac disease: From bench to bedside. Semin. Immunopathol..

[26] Di Sabatino A., Ciccocioppo R., Cupelli F., Cinque B., Millimaggi D., Clarkson M.M., Paulli M., Cifone M.G., Corazza G.R. (2006). Epithelium derived interleukin 15 regulates intraepithelial lymphocyte Th1 cytokine production, cytotoxicity, and survival in coeliac disease. Gut.

[27] Chander U., Leeman-Neill R.J., Bhagat G. (2018). Pathogenesis of Enteropathy-Associated T Cell Lymphoma. Curr. Hematol. Malign-Rep..

[28] Malamut G., El Machhour R., Montcuquet N., Martin-Lannerée S., Dusanter-Fourt I., Verkarre V., Mention J.-J., Rahmi G., Kiyono H., Butz E.A. (2010). IL-15 triggers an antiapoptotic pathway in human intraepithelial lymphocytes that is a potential new target in celiac disease–associated inflammation and lymphomagenesis. J. Clin. Investig..

[29] Schmitz F., Kooy-Winkelaar Y., Wiekmeijer A.-S., Brugman M.H., Mearin M.L., Mulder C., Lopes S.C.d.S., Mummery C.L., Staal F.J., van Bergen J. (2016). The composition and differentiation potential of the duodenal intraepithelial innate lymphocyte compartment is altered in coeliac disease. Gut.

[30] Schmitz F., Tjon J.M.-L., van Bergen J., Koning F. (2014). Dendritic cells promote expansion and survival of aberrant TCR-negative intraepithelial lymphocyte lines from refractory celiac disease type II patients. Mol. Immunol..

[31] Moffitt A.B., Ondrejka S.L., McKinney M., Rempel R.E., Goodlad J.R., Teh C.H., Leppa S., Mannisto S., Kovanen P.E., Tse E. (2017). Enteropathy-associated T cell lymphoma subtypes are characterized by loss of function of SETD2. J. Exp. Med..

[32] Cording S., Lhermitte L., Malamut G., Berrabah S., Trinquand A., Guegan N., Villarese P., Kaltenbach S., Meresse B., Khater S. (2021). Oncogenetic landscape of lymphomagenesis in coeliac disease. Gut.

[33] Vogel T.P., Milner J.D., Cooper M.A. (2015). The Ying and Yang of STAT3 in Human Disease. J. Clin. Immunol..

[34] Roberti A., Dobay M.P., Bisig B., Vallois D., Boéchat C., Lanitis E., Bouchindhomme B., Parrens M.C., Bossard C., Quintanilla-Martinez L. (2016). Type II enteropathy-associated T-cell lymphoma features a unique genomic profile with highly recurrent SETD2 alterations. Nat. Commun..

[35] Manso R., Rodriguez M., Chamizo C., Pérez N., Alonso-Alonso R., Minguez P.A., Borregon J., Baez-Duran E., Pozo E.M.C., Piris M.A. (2021). Intestinal T-Cell Lymphomas: Molecular Integrative Analysis Recognizes Different Therapeutic Targets for Each Subtype. Hematol. Oncol..

[36] Gooderham M.J., Forman S.B., Bissonnette R., Beebe J.S., Zhang W., Banfield C., Zhu L., Papacharalambous J., Vincent M.S., Peeva E. (2020). Efficacy and Safety of Oral Janus Kinase 1 Inhibitor Abrocitinib for Patients With Atopic Dermatitis: A Phase 2 Randomized Clin-ical Trial. JAMA Dermatol..

[37] Bao X., Ren T., Huang Y., Ren C., Yang K., Zhang H., Guo W. (2017). Bortezomib induces apoptosis and suppresses cell growth and metastasis by inactivation of Stat3 signaling in chondrosarcoma. Int. J. Oncol..

[38] Nicolae A., Xi L., Pham T.H., Pham T.-A., Navarro W., Meeker H.G., Pittaluga S., Jaffe E.S., Raffeld M. (2016). Mutations in the JAK/STAT and RAS signaling pathways are common in intestinal T-cell lymphomas. Leukemia.

[39] Clarke L., Adduri R.S., Smyth P., Quinn F., Jeffers M., Dunne B., O’Leary J., McKiernan S., Vandenberghe E., Pyne S. (2018). Potentially important miRNAs in enteropathy-associated T-cell lymphoma pathogenesis: A pilot study. Leuk. Res. Rep..

[40] Vaira V., Gaudioso G., Laginestra M.A., Terrasi A., Agostinelli C., Bosari S., Di Sabatino A., Vanoli A., Paulli M., Ferrero S. (2020). Deregulation of miRNAs-cMYC circuits is a key event in refractory celiac disease type-2 lymphomagenesis. Clin. Sci..

[41] Bhansali R.S., Barta S.K. (2023). SOHO State of the Art Updates and Next Questions|Challenging Cases in Rare T-Cell Lymphomas. Clin. Lymphoma Myeloma Leuk..

[42] Veloza L., Cavalieri D., Missiaglia E., Ledoux-Pilon A., Bisig B., Pereira B., Bonnet C., Poullot E., Quintanilla-Martinez L., Dubois R. (2023). Monomorphic epitheliotropic intestinal T-cell lymphoma comprises morphologic and genomic heterogeneity impacting outcome. Haematologica.

[43] Wang M., Yu M., Kong W.-J., Cui M., Gao F. (2021). Association between intestinal neoplasms and celiac disease: A review. World J. Gastrointest. Oncol..

